# The Effect of Time and Wear on the Human Teeth*Read before the Toronto Dental Society, February 25, 1918, and reprinted from *Oral Health* for April.

**Published:** 1918-05

**Authors:** A. E. Webster


### THE EFFECT OF TIME AND WEAR ON THE HUMAN TEETH *

BY A. E. WEBSTER, M.D., D.D.S., L.D.S.

The color of the enamel of the human teeth gradually darkens with years, but to a much less extent than is commonly supposed. It is the dentin which gives the general color to the teeth. The enamel, however, is subject to the influence of staining substances applied locally. In many parts of the world teeth are stained black or brown with vegetable stains. The betel nut is most commonly used for this purpose. Tobacco gives the enamel a distinct yellow hue. Enamel which readily becomes stained likely has some physical defect. Occasionally, enamel, which was perfect before a long, serious illness, becomes mottled yellow and white. A few cases have been seen in which the whole surfaces of the teeth are chalk white or dead white like paper. Such enamel may readily take

*Read before the Toronto Dental Society, February 25, 1918, and reprinted from *Oral Health* for April.

up stains and can be almost as readily destained. Old teeth, whose surfaces are much worn, stain more than those not worn.

Histologists have said that the enamel rods, as they approach the dentin, are more interlaced than at the surface, but this would not seem to be the case, judging from practical experience because enamels, whose surfaces are worn away, split more readily than the reduced thickness would seem to indicate. It is the loss of the cementing substance of the enamel which makes the chief changes in the color and in the friability. If the cementing substance is lost the teeth are a dead white, but may soon become stained from acids formed in the mouth or from vegetable stains. One often sees deciduous teeth much stained, especially if they have been considerably worn or too long retained.

Stained enamel may be destained by applications of hydrogen peroxide under heat as described by (Head). A pledget of cotton is saturated with peroxide of hydrogen and placed upon the surface of the enamel and a large hot instrument is placed upon it. This is repeated several times. Unfortunately destained teeth soon return to their former color because the enamel is defective or porous and the cause of the original staining is likely still present.

There is a type of pink tooth which occasionally occurs in one or more of the anterior teeth that must not be confounded with the pink tooth following the devitalization of a pulp, or the pink hue due to a staining of the enamel. The pink tooth mentioned seems to have the color in the dentin.

The wise dentist will base his treatment of discolored enamels upon the cause and the prognosis, knowing that as years go on pigmentations increase. There are green stains on the surface of the anterior teeth of young people and reddish pigmentations on the surface at the gingiva, found in some localities which are not to be considered at this time.

As years pass, the enamel becomes gradually thinner and thinner, depending upon the amount of grit or friction applied to the surface. Among those races of people who use sand on wood fibre sticks to polish their teeth, the enamel is frequently worn completely through. This also occurs among people who live in sandy desert places or among those who eat foods of a sandy character. Chewing tobacco will do it. As the enamel becomes thinner it becomes more friable, having checks or cracks almost the full length of the crown. These checks are often open wide enough to admit staining substances, thus giving a yellow streaked appearance to the labial enamel. Dentists of experience have often been struck by the ease with which enamel will check when sharp instruments accidentally cut across its surface. Corners of incisors seem to split off without much apparent reason. Such enamel needs special attention in cavity preparation; namely, lap joint inlays. If the pulp is lost there is no doubt about the enamel becoming more and more friable as years pass. Such enamel is less strong and should receive different treatment in cavity preparation from the enamel of vital teeth.

Besides the wear from abrasion there is the wear of attrition and erosion. Some enamels and dentines, especially of the deciduous teeth, wear away until the half of the substance of the teeth is lost. A peculiar thing about some of this wear is the cupping of the occlusal surface. It would seem that as soon as the enamel is worn through, the dentin hollows out, though it does not come in contact with an antagonizing tooth. An erosion rather than an attrition or abrasion. These cuppings are often so deep that fruit seeds and other foods wedge into them splitting the surrounding enamel. Judicious grinding of the enamel, in such cases, will avoid large plates of enamel being split off. It is not at all uncommon to see the teeth of old people worn to the gum line, especially is this the case in the anterior part of the mouth, if the molars have been lost.

The effect of occlusal wear, if in marked degree, is to bring the upper and lower jaws closer together with the consequent change in the condyle path. With the closure of the jaws there is a protrusion of the lower. If the molars and biscuspids have been lost, an edge to edge bite is developed which usually brings about a rapid wear with less cupping. In some cases the upper anterior teeth are protruded and worn chiefly on the lingual aspects. Teeth which once antagonized normally will sometimes develop a gliding wear which will place them in false positions, while in other cases, the wear will be such as to lock the bite, giving the patient no possibility of lateral movement while the teeth are in apposition. Single teeth often begin to change position without any apparent periclasia, such cases often have their origin in occlusal wear. Such mal-occlusions as are developed from occlusal wear might be classified as traumatic occlusions and undoubtedly have a baneful influence on the peridental membrane. Having recognized the condition and its prognosis if untreated, the dentists' duty is clear. Restore the teeth and occlusion to the normal for this patient, keeping in mind that which is normal for one patient is not normal for all others. Don't be afraid to use carbon paper and the stone but leave all ground surfaces highly polished.

It would seem that the more rapid the occlusal wear, the more likelihood of the surfaces being sensitive to attrition. If acid fruits are indulged in, such exposed dentines become exceedingly sensitive. In cases of ebornation, however, no such sensitiveness develops from the use of acid fruits or become sensitive to thermal changes. Pathological pulps are undoubtedly developed under worn teeth as hyperaemic pulps, pulp stones, atrophic pulps and marked pulp recession are more likely to be met with than in unworn teeth. If for no other reason than to save the develoment of pathological pulps, exposed dentin should be protected from irritation. One's observation should have told us that the tooth tissues are exceedingly porous, but it remained for Bunting to demonstrate it. In future exposed dentines will get more attention than in the past.

Erosions, which are commonly supposed to be found on the labial, buccal, and occasionally, the lingual surfaces are in the writer's opinion not at all different from the loss of tissue which occurs on the occlusal surface. There is undoubtedly a combination of solution and attrition or abrasion. The tooth tissue is softened by a weak acid and then the tooth brush or food readily wears away the surface. All the peculiar notchings of the labial and buccal surfaces can be readily reproduced by artificial brushings. Once the enamel is worn through the dentin goes rapidly.

Dental toilet preparations, such as pastes and powders, depend upon a grit which they contain for their value. Some of these preparations contain so much grit that if used once or twice a day over a period of many years, the enamel will be worn through and deep cuts made into the dentin. Those preparations which have a finely ground powder are not as efficient for cleaning the surfaces of the enamel and may be used much more frequently. It is essential that a dentist, in recommending any tooth preparation, should also know how frequently it should be used.

In many cases of considerable occlusal wear in the molar and bicuspid region, contact is lost and the patient is unable to masticate satisfactorily, although he may have a full complement of teeth.

The writer has observed that teeth which wear away badly rarely have dental caries and what is more, such dentines cut easily as if they were less dense. If for some-reason, solution ceases, then there develops a hard glistening surface as if brought about by burnishing. This surface is often as hard as some enamels. It is called ebornation or tubular calcification.

The linguo-occlusal surfaces of incisors wear away until the dentin is exposed, leaving the thin labial plate of enamel unsupported by dentin which readily splits off leaving a saw edge to the enamel or in some cases so much splits off as to leave distinct notches. Such wearing and notching is more rapid if the molar teeth have been lost.

When teeth have been lost or there is a marked mal-occlusion there is often a gliding wear which cuts deeply into the tooth structure, causing the recession of the pulp, or its death, as well as peridental irritation.

Dentists of experience and observation have recognized that natural tooth tissue wears away more rapidly than fillings, inlays, crowns, bridges or artificial teeth, but how many have applied this knowledge so as to prevent the failure of former dental operations. As the teeth wear down, fillings, inlays, crowns and bridges receive more and more of the weight of occlusion. All have recognized, with pride, the glistening burnished spot on the lingual surface of an anterior filling or inlay, showing the heavy occlusion delivered upon this surface. Proud of our former skill because the filling has not been dislodged, though it receives unusual stress. The consequence of such impact is certain to dislodge the filling and, perhaps, the corner of the tooth as well. If it should happen to be a crown, it is sure to bend the pin or split the root. Such points of weight of occlusion should be freed by grinding from time to time, as necessity demands. This is a prophylactic measure. How we have watched that large pure gold inlay or amalgam filling being beaten by an antagonizing molar tooth as if it were intended to make gold foil of it. Sooner or later such fillings will fail. An amalgam so beaten will flow, if the filling doesn't fail, the peridental membrane will.

Another consequence of occlusal and proximal wear is the development of periclasia from wedging of food and traumatic occlusion. Since the introduction of the inlay process, there is no reason for not protecting against occlusal wear and proximal wear as well.

There is an approximate wear of the teeth which is little recognized and is always associated with marked occlusal wear. The individual movement of the teeth is sufficient to wear distinct facets on the proximal surfaces thus leaving flat contacts where there were once convex contacts. This reduces the mesio-distal diameter of the teeth and permits food to pass between them and makes the beginning of periclasia.

The wear of artificial appliances in the mouth will not be considered in this connection.

Time undoubtedly brings about a certain chemical change in both the enamel and the dentin as occurs in other hard structures of the body. This chemical change has its chief effect on the cementing substance of the enamel rods. There is a reduction of those vital elements which hold the rods together and an apparent increase of the calcium carbonate elements. Though enamels may be more resistant to cutting instruments they split easier as years go on. So it is with dentin, there is an increase of the calcium salts under normal conditions and yet there would seem to be a distinct decrease in these elements under pathological influences. Every operator has recognized that tooth tissue gradually becomes less sensitive to cutting instruments as years go by, and that pathological dentin or the pulp of the old patient may be more sensitive than that of the young.

These chemical changes in the structure of the teeth must not be looked upon as being sufficient to make the tooth of the old person less liable to dental caries than the young. The cause is much more obscure than this.

Having directed our attention in a very cursory manner to what occurs in time and wear of human teeth, let us now think of the effects of those changes. How should the knowledge of these things influence our practice? It is all very well for us to examine a young patient's mouth and recognize several small gold fillings in the proximal surfaces of the anterior teeth, and also a slight linguo-occlusal wear, but how many of us really begin to figure out what this means for the patient twenty-five or forty years hence? It is one thing to discover what is now happening, but unless we are fully aware of the remote consequence of the present conditions we are not likely to apply appropriate prophylactic measures.

The time was when dentists were most anxious to find a remedy for the symptoms presented by their patients while today many are keenly exercised about the cause of the symptoms or the diagnosis. This is good as far as it goes, but let us now look into the future a little further and study the remote consequence of a wearing down molar or a decay at the gingival of a second molar, at sixteen years of age. How unconcerned we are when a child of ten or twelve presents with a beginning caries in the upper lateral incisor. Let me remind you older practitioners lest you have forgotten, and sound a warning to the younger men of the dental history of such a lateral incisor. A cement filling for two years. A silicate for perhaps three more, or perhaps a small gold filling for the same time, or perhaps five years more. Each succeeding filling getting larger and larger until the occlusion strikes heavily upon the lingual surface, as the teeth wear away. The filling receives the occlusion and is dislodged together with the corner of the tooth. Now a step cavity with either a gold filling or an inlay, this lasts well, until, perhaps, the molars and bicuspids are lost or the teeth wear away until occlusion again dislodges the filling or a few months' neglect has caused the involvement of the pulp. The pulp is devitalized, the root is filled, another inlay or crown is placed. In a few years more the patient develops rheumatism, endocarditis, chronic Bright's or gastric ulcer. If the root is comfortable, it may not be suspected. An accidental heavy pressure splits it, when no further mechanical arts can do anything for it except extract it. The extraction relieves the patient of her chronic systemic disease. A marvelous cure made by Dr. Brown or somebody's pink pills for pale people. The patient blames the dentist for splitting the root when the crown was being set, and the dentist blames the patient for cracking nuts with her teeth; none of them recognizing that the splitting of the root was a godsend and that the original caries was the serious misfortune. This is but a short history of a small cavity in the lateral incisor.

Who among us thinks over the future dental history of each tooth, as well as the teeth of our patients, as we serve them from time to time? Until we can predict the dental future of our patients we will be unable to guide them through a long life with the best possible results. So long as we continue to treat the needs of our patients from day to day, so long should we be classed as tinkers having no thought for the morrow.

If we are now convinced of the importance of looking into the future dental history of our patients, what are the effects of the wear of the teeth upon the occlusion, the facial contour, the pulp, our former dental operations and the peridental membrane, on the soft tissues and what prophylactic remedies may be instituted to, as far as possible, minimize any baneful consequences?

Before a body of this kind, it is not necessary to do more than point out the necessity and direct the way and the rest will follow. Preventive measures based upon a knowledge of the structures involved, the forces at work and the history of many cases gone before, will do much for the dental future of our patients' comfort and health, as well as great good to the profession.

If someone would write the dental history of several patients it would awaken in the minds of the profession a realization of the shortcomings of their calling which would forever do away with such expressions as permanent denture, permanent fillings, guaranteed crowns, fillings, etc.—*Oral Health.*

